# Garlic alters the expression of putative virulence factor genes *SIR2* and *ECE1* in vulvovaginal *C. albicans* isolates

**DOI:** 10.1038/s41598-020-60178-0

**Published:** 2020-02-27

**Authors:** Mohamed M. Said, Cathy Watson, Danilla Grando

**Affiliations:** 1 School of Science, RMIT University, PO Box 71, Bundoora, 3083 Australia; 2 School of Science, Al Zintan University, Al Zintan, Libya; 3 Department of General Practice, The University of Melbourne, 3rd Floor, 780 Elizabeth St, Carlton, VIC 3010 Australia; 4 Department of General Practice, Monash University, 1/270 Ferntree Gully Rd, Notting Hill, VIC 3168 Australia

**Keywords:** Clinical microbiology, Fungal biology

## Abstract

Vulvovaginal candidiasis causes sufferers much discomfort. Phytotherapy with garlic has been reported to be a possible alternative form of treatment; however, it is unknown why patients report varying success with this strategy. Fresh garlic extract has been shown to down-regulate the putative virulence gene, *SIR2* in *C. albicans*. Our study aimed to see if previous observations were reproducible for the gene responsible for Candidalysin (*ECE1*). Two clinical strains from patients with reported variable efficacy of using garlic for the treatment of vulvovaginal candidiasis were compared through biofilm assays and antimicrobial susceptibility. Real-time PCR was used to assess changes in gene expression when exposed to garlic. Treatment with fresh garlic extract and pure allicin (an active compound produced in cut garlic) resulted in a decrease in *SIR2* expression in all strains. In contrast, *ECE1* expression was up-regulated in a reference strain and an isolate from a patient unresponsive to garlic therapy, while in an isolate from a patient responsive to garlic therapy, down-regulation of *ECE1* occurred. Future studies that investigate the effectiveness of phytotherapies should take into account possible varying responses of individual strains and that gene expression may be amplified in the presence of serum.

## Introduction

Some virulence factors thought to be responsible for the pathogenicity of *C. albicans* are expressed when a suitable environment exists to cause disease. These factors include those that enable attachment of *C. albicans* to the site of infection (adhesion). Additionally, *C*. *albicans* undergoes a morphogenesis process which is a reversible transformation between the unicellular form (yeast) and the pathogenic filamentous form (hyphae). This morphogenesis process is thought to herald the symptoms of itchiness and result in discharge due to the localised inflammatory response in vulvovaginal candidiasis (VVC). Blostein *et al*.^1^ estimated that approximately 40% women will experience uncomplicated vaginal candidiasis by the time they have reached 50 years of age and a significant number of these women (up to 20%) will suffer recurrent vulvovaginal candidiasis (RVVC), which is defined as four or more symptomatic episodes of VVC in 12 months. Sobel *et al*.^2^ proposed long-term therapy for RVVC which is expensive, with up to 50% of women relapsing after therapy ceases. Side-effects, such as headaches and gastrointestinal disturbances, were reported. Because of challenges in managing RVVC, many women seek alternative types of therapies such as phytotherapies^3^. Allicin, a compound formed on the cutting of the garlic clove (*Allium sativum*), has antifungal activity^4^ and has been used by women to bring symptomatic relief in VVC^5^.

*C. albicans* has several virulence genes that play an essential role in its pathogenicity. It has previously been reported that fresh garlic extract (FGE) down-regulates the silent information regulatory gene (*SIR2*) in *C. albicans* ATCC 14053^6,7^. Since the report of garlic down-regulation in *SIR2*, an additional virulence gene *ECE1* (extent of cell elongation) has been shown to be important in one of the crucial steps to the commencement of infection (attachment) and is highly expressed during the invasion of host tissue and during epithelial infection^8,9^. It has recently been reported that the post-translational processing of *ECE1* results in the production of the first described cytotoxin for *Candida* spp. called Candidalysin^10^. Candidalysin has been demonstrated to be responsible for the immunopathogenesis of *C. albicans* vaginitis^11^. These results suggest that *ECE1* is an essential virulence gene of *C. albicans* expressed during the invasion of host cells. The ability of garlic to affect the transcription of this gene has not been reported.

One of the difficulties when undertaking studies to determine the efficacy of treatments for VVC is that a laboratory measure of treatment success used is the reduction in colony forming units of yeast derived from vaginal swabs^12^. In this previous study we investigated the use of oral garlic tablets (Garlicin™) for the treatment of VVC. A demonstrated therapeutic response was achieved by some women, however this study was not sufficiently powered to detect a statistically significant difference between colony counts of candida in women taking garlic and those not taking garlic^12^. Measuring colony counts may not reflect that symptoms may result from morphogenic switching from the yeast to hyphal form. It may be possible that women who experience an amelioration of symptoms when taking garlic have less switching of yeast to the filamentous form.

In the present study we wished to investigate whether there was evidence that the differing efficacy of garlic treatment observed may be dependent on the *Candida* strain. Garlic extract was used to test its effect on the transcription of two genes, the previously reported *SIR2* as a control gene and *ECE1*, using real-time PCR. We also wished to investigate whether the testing milieu was important as biological substances such as serum are known to enhance the switching of blastospores to the more invasive hyphal form^13–16^. At the same time, serum has been shown to have an inhibitory effect on the formation of biofilm in some bacteria such as *Staphylococcus aureus*^17^ and *Pseudomonas aeruginosa*^18^. It is unknown what effects fetal bovine serum (FBS) has on *Candida* biofilm. Wild-type *C. albicans* cells have been reported to have improved survival and growth when grown in the presence of FBS^19,20^. Thus we also wished to investigate whether differences in response to garlic therapy could be attributed to biofilm formation. Finally we wished to determine whether FBS also affected measurements of *ECE1* expression.

## Results

### Broth microdilution method for determination of MIC

The MIC50 and MIC90 are presented in Table 1. Although the MIC for the ATCC strain for fluconazole was higher than the VVC clinical strains, it was still lower than the breakpoint for resistance as reported by Cortés *et al*.^21^.

**Table 1:** MIC50 and 90 of isolates as determined by broth microdilution assay.

Strain and isolates	Fresh garlic extract	Fluconazole
	MIC50/MIC90 (mg/ml)	MIC50/MIC90 (µg/ml)
*C. albicans* ATCC 14053	0.05/0.4	2/4
*C. albicans* isolate 0861	0.05/0.4	0.2/2
*C. albicans* isolate 1358	0.05/0.4	0.2/2

### Investigation of the effect of different concentrations of FGE on biofilm formation of C. albicans with and without 3% fetal bovine serum (FBS)

**Figure 1:**
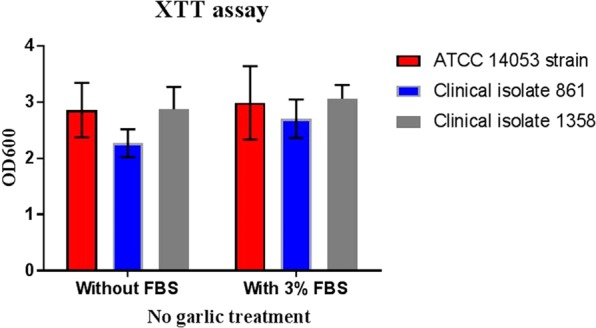
Distribution of *C. albicans* ATCC 14053 and clinical isolates 0861 and 1358 biofilm without garlic treatment in the presence and absence of 3% FBS. Bars indicate standard error of the mean (SEM).

The amount of biofilm produced by the strains studied in shown in Fig. 1. Although strain 0861 (clinically responsive to garlic therapy) produced less biofilm, this amount was not statistically different to the other two strains tested.

**Figure 2:**
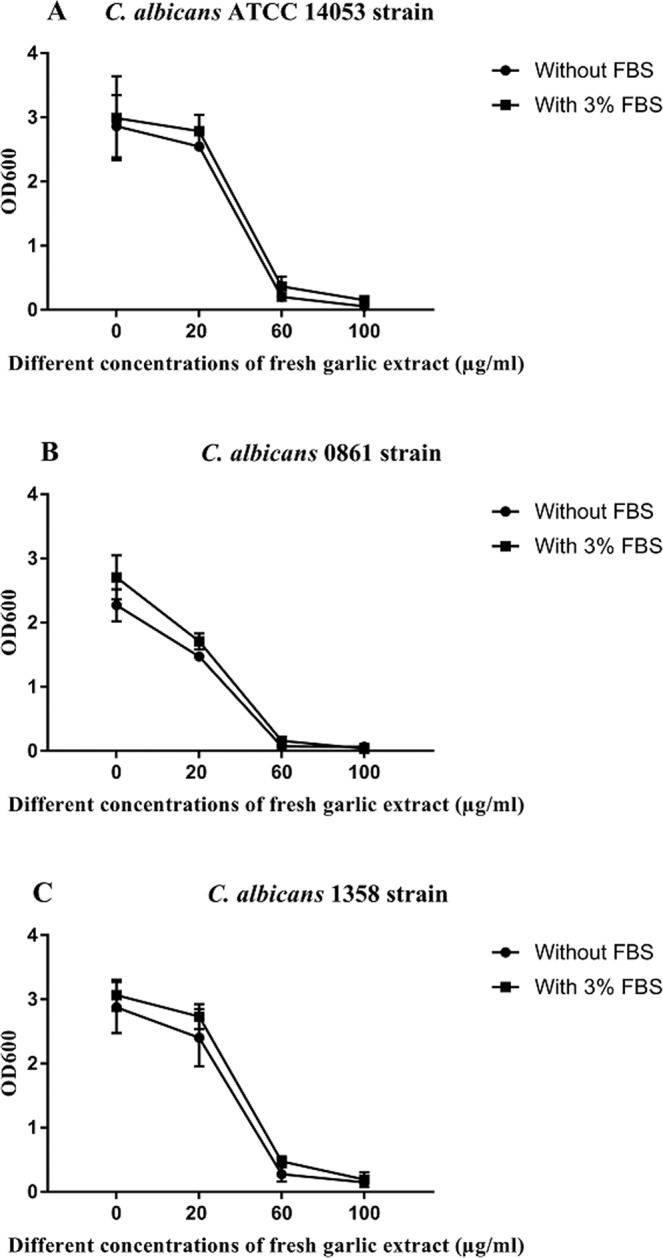
Distribution of *C. albicans* ATCC 14053 (**A**) and clinical isolates 0861 (**B**) and 1358 (**C**) biofilm at different concentrations of FGE in the presence and absence of 3% FBS. Bars indicate SEM.

The inhibitory effect of fresh garlic extract on biofilm formation of the strains was demonstrated over a range of FGE concentrations with and without FBS. There was no difference seen in the kinetics of adherence and subsequent biofilm formation in the strains tested (Fig. 2). The reduction in biofilm in the presence of garlic at levels of 60 μg/ml and above was statistically significant (*P* < 0.001). Statistical analysis of the OD of the three isolates in the presence of garlic with and without FBS using two-way ANOVA showed no significant difference in the biofilm formation between these three isolates.

### Effect of FGE and allicin on *C. albicans* gene expression

**Figure 3:**
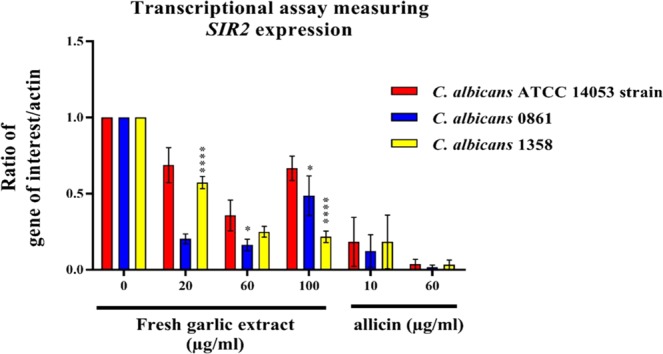
Relative quantitation of *SIR2* expression normalized to the *β-actin* in *C. albicans* ATCC 14053 and clinical isolates 0861 and 1358 planktonic cells after 24 h of treatment with different concentrations of FGE and pure allicin by RT-PCR. Untreated samples of the three isolates were used as a reference to show the differences among strains. The * denotes significant reduction of gene expression to untreated control at levels ranges between *P* < 0.05 (*) and *P* < 0.0001 (****). Data shows the ratio of gene expression with bars indicating SEM.

The relative quantification of *SIR2* and *ECE1* expression ratio normalized to *β-actin* indicated that both genes tested had significant differences in gene expression between the antifungal exposed cells and the untreated control. A general trend of reduction in *SIR2* expression was seen in all isolates tested and exposed to FGE compared with cells that had not been treated with FGE and pure allicin (Fig. 3). At 20 µg/ml and 60 µg/ml FGE there were significant reductions of *SIR2* expression; however, this was not as significant at 100 µg/ml. This difference is because the growth of cells is severely reduced at 100 µg/ml of FGE, expression is at low levels and thus results should be interpreted with caution. The results of allicin testing suggests that the effect of 10 µg/ml of allicin is equivalent to 60 µg/ml of FGE. 60 ug/ml of allicin had an even more significant effect than 10 µg/ml, while not showing the effect seen by 100 µg/ml of FGE. The clinically resistant isolate 1358 behaved similarly in *SIR2* expression except for perhaps activity of the gene was still noticeable at 100 µg/ml. The reduction in *SIR2* expression at 20 µg/ml FGE was most marked in the clinically responsive isolate 0861. The clinically resistant isolate 1358 was significantly less affected in its *SIR2* expression compared to clinical isolate 0861 (*P* < 0.001). At 60 µg/ml FGE, isolate 0861 was significantly different to the ATCC strain (*P* < 0.05); however, isolate 1358 was not significantly different.

**Figure 4:**
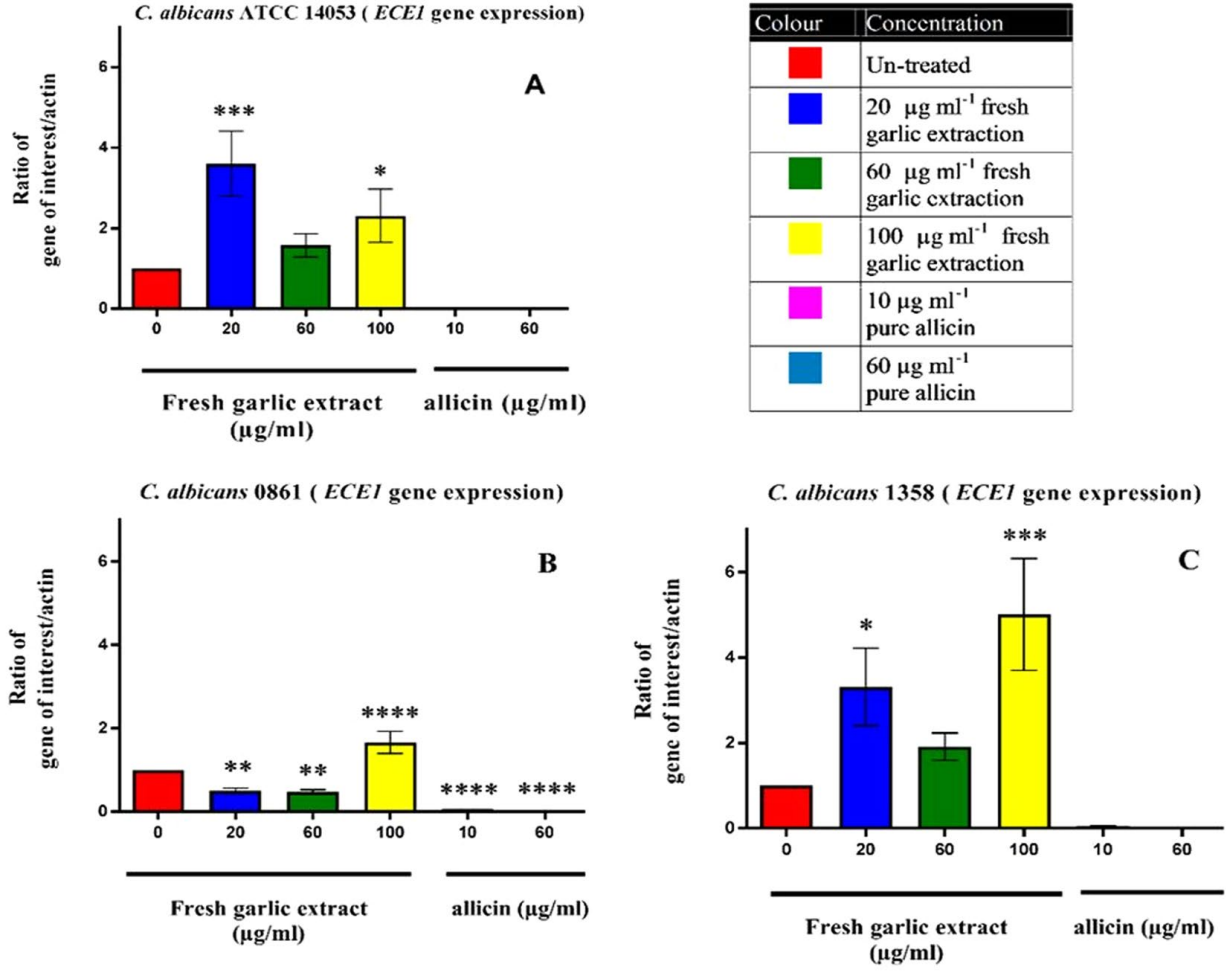
Relative quantitation of *ECE1* expressions normalized to *β-actin* in *C. albicans* ATCC 14053 (**A**) and clinical isolate 0861 (**B**) and 1358 (**C**) in planktonic cells after 24 h of treatment with different concentrations of FGE and pure allicin showing a significant reduction of gene expression in clinical isolate 0861 while up-regulation is shown in ATCC strain and clinical isolate 1358 at all concentrations used compared to untreated control. The * denotes significant reduction of gene expression to untreated control at levels ranges between *P* < 0.05 (*) and *P* < 0.0001 (****). Data shows the ratio of gene expression with bars indicating SEM.

In contrast, the behaviour of *ECE1* expression was markedly different from the *SIR2* gene. The results are shown in Fig. 4(A–C). There is an increase in *ECE1* expression in cells exposed to FGE compared to non-exposed cells. FGE has an up-regulation effect on the *ECE1*. On the other hand, the normalised *ECE1* expression level for cells incubated with pure allicin showed a complete reduction in the expression of *ECE1*. Down-regulation of *ECE1* in the clinically responsive isolate 0861 was markedly different to the upregulation of the gene seen in the clinically unresponsive isolate 1358.

### Effect of FBS on ECE1 expression in *C. albicans* biofilm

**Figure 5:**
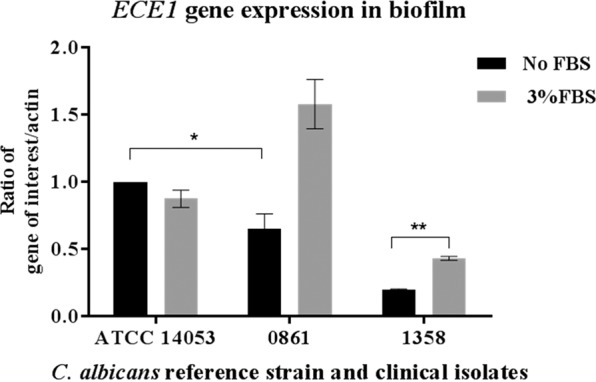
Relative quantitation of *ECE1* expression normalized to the *β-actin* gene in *C. albicans* ATCC 14053 and clinical isolates 0861 and 1358 biofilms after 24 h of treatment with and without 3% FBS. Untreated ATCC 14053 strain was used as a reference strain to show the differences among strains. (*) means a significant down-regulation (*P* < 0.05), (**) means a significant up-regulation of *ECE1* expression (*P* < 0.01). All other difference observed were significant at P < 0.001. Bars indicate SEM.

A quantitative relative real-time PCR assay was performed to measure the expression of *ECE1* in the *C. albicans* ATCC 14053 strain and clinical isolates 0861 and 1358 when treated with 3% FBS, including primers for the *β-actin* gene and amplified under the same conditions to normalize the expression of both genes. The *ECE1* mRNA expression for both clinical isolates was significantly different in the presence of FBS compared to the absence of FBS as shown in Fig. 5 (*P* < 0.001). In the clinically responsive 0861 strain, FBS reversed the down-regulation of *ECE1* compared to the ATCC strain being upregulated. There was a significant difference in gene expression between the two clinical isolates. If only the ATCC strain had been tested, then it would not have been thought necessary to include FBS in the cultural milieu. The presence of FBS has a significant impact on the expression levels seen in the additional isolates included in this study.

## Discussion

Garlic (*Allium sativum*) has been used throughout history as an herbal medicine. Previous studies performed *in vitro* have established the role of allicin as an antifungal agent against *Candida* spp., *Trichophyton* and *Aspergillus*^22^. In addition, a mouse model of systemic aspergillosis^23^ demonstrated the antifungal potential of allicin against *Aspergillus* spp. The present study examined two isolates from VVC patients differing in their response to garlic treatment. It would be important to also look at additional blood isolates such as the reference strain ATCC 14053 used in the present study to see if there is also varying transcriptional expression of virulence proteins in these isolates.

The endpoint MIC was determined to see if the clinical isolates had differing profiles in susceptibility testing to the ATCC strain 14053. The results of MIC testing showed that this could not explain the differing responses to garlic therapy experienced by the two patients selected from the study performed by Watson *et al*.^12^. The effect of garlic on the strains tested was measured by both MIC determination and biofilm formation. Khodavandi *et al*.^24^ reported that for ATCC 14053, the MIC for FGE was 0.1 mg/ml and for Fluconazole, 2 µg/ml. In the present study, the MIC of 0.4 mg/ml FGE for the ATCC 14053 strain was lower than that observed by Ghannoum^25^ (2 mg/ml) and Lemar *et al*.^26^ (10 mg/ml) but slightly higher than Khodavandi *et al*.^24^. This variation is probably due to methodological issues such as preparation time and time until use. Thus it was decided to also include allicin (one of the commercially available pure bioactive components of cut garlic clove) in the transcriptional assays as a standard. During MIC testing, Fluconazole was used as quality control for the MIC method and the results were within acceptable limits^27^. In our study, we observed that 0.05 mg/ml of FGE was able to produce a partial inhibitory effect (MIC50) for all our strains. The MICs of FGE were 0.4 mg/ml and for Fluconazole 0.2 µg/ml (both clinical isolates) and 4 µg/ml (ATCC strain), which for the ATCC strain, is similar to findings from previous reports^28,29^. Thus these isolates were sensitive to fluconazole and no differences in drug susceptibility were seen that could explain the different clinical responses in the two isolates.

The colonization of *Candida* to the surfaces of various tissues is the precursor to symptomatic infection. Also, biofilm formation is one of the mechanisms used by *Candida* spp. to resist the treatment with antifungals by forming a barrier to them^30^. In this study, the clinical isolates 0861 and 1358 were compared to the control strain ATCC 14053 with regard to biofilm formation when treated with different concentrations of FGE in the presence and absence of 3% of FBS. Our data showed a trend of reduction in biofilm formation in all isolates treated with 60 and 100 µg/ml of FGE in the presence and absence of 3% FBS as measured by the XTT assay. The presence of serum did not influence these results. These results indicate that biofilm production was not the cause of the different clinical responses in our two selected patients.

In earlier reports *SIR2* was thought to be a key gene in the phenotypic switching in *C. albicans*^31^. This led to investigations that showed the down-regulation of this gene in the ATCC 14053 strain as reported by Khodavandi *et al*.^24^ and Low *et al*.^7^. Our findings here confirm the significant inhibition in *SIR2* expression relative to a housekeeping gene in the *C. albicans* ATCC 14053 strain as well as clinical isolates 0861 and 1358 when treated with more than 20 µg/ml FGE and more than 10 µg/ml of pure allicin. *SIR2* is no longer considered central to the process of hyphal formation^32^. Thus in the present study it was important to investigate a gene now considered to be central to hyphal formation, *ECE1*^32^.

*ECE1* is highly expressed during the transformation from yeast form to hyphae form^33^. In the present study it showed significant down-regulation relative to the housekeeping gene in the clinical isolate responsive to garlic therapy (0861) when treated with 20 and 60 µg/ml of FGE and 10 and 60 µg/ml of pure allicin. In contrast, up-regulation was shown in the ATCC 14053 strain (an isolate originally derived from a patient’s blood) and the clinically unresponsive isolate 1358 when treated with the same concentrations of FGE, but not with pure allicin. Interestingly isolate 0861 shows a reversal in regulation at the higher level of 100 µg/ml although it is still significantly reduced from untreated cells. This was also seen in the ATCC strain but not in the clinically unresponsive 1358 isolate. Differential expression of *ECE1* may explain how the cells in the clinically unresponsive isolate were possibly able to persist and increase the inflammatory mediator Candidalysin, thus symptoms continued in this patient.

Finally, as *ECE1* is central to hyphal formation and hyphal formation is influenced by the presence of serum, it is interesting to see the effect of fetal bovine serum on *ECE1* expression. This final experiment showed a significant increase in *ECE1* expression in induced biofilm exposed to FBS. This may indicate that serum may better simulate *in vivo* conditions and be important to add in future assays when determining the effects of putative phytotherapies on the transcription of important virulence genes.

## Conclusion

In conclusion, this pilot study provides evidence that response of *Candida albicans* to a complementary medicine such as garlic may be strain dependent. Our pilot study also showed upregulation of *ECE1* in a clinical isolate unresponsive to garlic therapy and downregulation of the gene in a patient responsive to garlic therapy. *ECE1* expression was suppressed in all strains when treated with pure allicin. These results, although preliminary in nature, indicate that there may a role for garlic and its derivatives to ameliorate the symptoms of VVC through the downregulation of important virulence genes. These strain dependent characteristics can be identified *in vitro* but may be dependent on the testing milieu. If our hypothesis is correct, then a personalised medicine approach may be needed to determine when garlic can effectively be used in the management of VVC. This finding has broader implications as antibodies to ECE1 have previously been demonstrated in the serum of patients with invasive candidiasis^34^, and thus complementary medicine such as garlic may have usefulness beyond the treatment of mucosal candidiasis.

## Materials and Methods

### Strains, media and inoculum preparation

*C. albicans* ATCC 14053 and two *C. albicans* clinical isolates 0861 and 1358 previously isolated and identified by Watson *et al*.^12^ were tested. These strains were chosen as 0861 was isolated from a patient who reported symptomatic relief from taking garlic as a complementary medicine while 1358 was isolated from a patient who did not report any change in the typical candidiasis symptoms experienced. The isolates were first subcultured on sabouraud dextrose agar (SDA) and incubated for 24 h at 35 °C and passaged at least twice to ensure purity and viability. Stock inoculum suspensions of the *Candida* spp. were prepared by selecting five colonies from 24 h cultures grown on SDA at 35 °C and suspending in 5 ml of sterile saline (0.85%) v/v NaCl. Cell density was adjusted with a spectrophotometer at 530 nm wavelengths to achieve a turbidity equivalent of a 0.5 McFarland standard. Each sample was maintained in 20% (v/v) sterile glycerol stocks and subcultured on yeast-nitrogen base with amino acids at 35–37 °C for 24–48 h to ensure viability and purity before testing.

### Antifungal agents

Garlic extract was prepared freshly (FGE) before each test according to Lemar *at al*.^26^ from peeled and crushed garlic (*Allium sativum*) cloves sourced from a local retail outlet. A total weight of 10 g of fresh garlic was ground in 10 ml of distilled water to prepare a stock solution of 1 g/ml. Serial dilutions were prepared from the stock solution to give concentrations of 20, 60 and 100 µg/ml. The solution was allowed to stand for 30 minutes at room temperature followed by centrifugation at 4500 x *g* for 10 min. The supernatant fluid was then passed through a sterile filter (0.22 µm). As FGE is a crude extract, we wished to include for our transcriptional assays a chemically defined compound derived from garlic. Commercial allicin solution (Sapphire Bioscience Pty. Ltd., Australia) was prepared according to the supplier’s recommendation and kept at −80 °C until use. Fluconazole (Sigma Chemicals Co. St. Louis, MO, USA) solution was prepared according to the manufacturer’s instructions (5 mg/ml) by dissolving in dimethyl sulfoxide and stored frozen at −70 °C until use.

### Broth microdilution method for determination of MIC

Microdilution broth susceptibility testing was performed in triplicate according to the CLSI M27 Reference Method for Broth Dilution Antifungal Susceptibility Testing of Yeasts^27^ and the growth kinetics of isolates were measured by spectrophotometry at 530 nm. The concentrations of fluconazole tested were 0.125–64 µg/ml. The concentrations of fresh garlic extract tested ranged from 0.025 mg/ml to 25 µg/ml. The control strain *Candida albicans* ATCC14053 and its response to fluconazole was used for quality control. The lowest drug concentration that resulted in a 50% reduction in growth (measured by spectrophotometry) compared to the control well was called the MIC50 and the concentration that resulted in no visual growth was called the MIC90. These scores were used to determine if there were any discernible differences in how the clinical isolates responded to the antimicrobials.

### Effect of FGE on biofilm formation in the presence and absence of 3% FBS

#### Establishment of biofilms

*C. albicans*, reference strain 14053 and the two VVC clinical isolates (0861 and 1358) were induced to form biofilm in the presence and absence of 3% FBS according to the protocol of Braga *et al*.^35^ with slight modifications. Briefly, a suspension of each isolate containing 1 × 10^6^ cells/ml was added to 100 µl of different concentrations of FGE (20, 60 and 100 µg/ml) using a tissue culture 96 well plate in the absence and presence of 3% FBS and incubated at 37 °C for at least 90 min (with no shaking). The experiments were performed in triplicate and included control untreated wells. Subsequently, the mixture was incubated at 37 °C for 24 h (growth phase) with gentle shaking^36,37^. The effect of antifungal agents on the biofilm formation by three different *C. albicans* strains was quantified using an XTT reduction assay as described below, according to Peeters *et al*.^38^.

#### Quantification of biofilm by XTT assay

In the biofilm quantification protocol using an XTT assay, 4 mg XTT (Sapphire Bioscience, Australia) in 10 ml prewarmed phosphate buffered saline (PBS) was dissolved and supplemented by 100 µl menadione stock solution, which contained 55 mg menadione (Sapphire Bioscience, Australia) in 100 ml acetone. One hundred µl of the XTT/menadione solution was added to all wells and incubated in the dark at 37 °C for five h. The contents of the wells were transferred to Eppendorf tubes and centrifuged at 13,000 rpm for four min. One hundred µl of supernatant from each well was transferred to new microplate wells and the absorbance measured using spectrophotometry at 600 nm (FLUOstar Omega microplate reader).

### Effect of FGE and pure allicin on *C. albicans* gene expression ECE1 and SIR2

Suspensions of the *C. albicans* isolates containing 1 × 10^6^ cells/ml in RPMI 1640 (with L-glutamine) were incubated with FGE at final concentrations of 0, 20, 60 and 100 µg/ml and pure allicin at final concentrations of 10 and 60 μg/ml, and then incubated at 37 °C for 24 h with brief shaking. The concentrations of FGE were chosen according to those used by Low *et al*.^7^. In order to measure mRNA, total RNA was extracted from a broth culture of *C. albicans* ATCC 14053 and clinical isolates using the RNeasy mini kit (Qiagen, Germany) with a slight modification for yeast. According to the manufacturer’s operating instructions for yeast cells, 2 ml of sorbitol lysis buffer (1 M sorbitol and 0.1 M EDTA pH 7.4) was added to 5–10 ml of broth culture of each treated sample. Then 50 U of lyticase (ICN Chemicals, USA), and 10 μl of β-Mercaptoethanol were added to the mixture to lyse the yeast cell wall and generate spheroplasts. The RNA solution obtained was then purified with Turbo DNA-free kit (Ambion Life Technologies, Carlsbad, CA, USA) to remove residual DNA. The RNA concentration and quality were measured for purity estimation using a POLARstar Omega. The ratios of readings of A260/A280 and A260/A230 for all samples were above 2.0 in nuclease-free water. Single-stranded cDNA was synthesised from approximately one μg of the resulting RNA using a QuantiTect Reverse Transcription Kit (QIAGEN, Australia). The reverse transcription reactions were performed in triplicate.

All primers in this study were designed for gene expression by locating them at start and stop codons using Clone Manager suite of analysis tools (Table 2). The accession number of *ECE1* was NC_032092 and that for *SIR2* AF045774. *β-actin* was used as a reference housekeeping gene. A negative control without QuantiTect reverse transcriptase was included for each sample to ensure that the PCR products originated from cDNA. *SIR2* and *ECE1* genes from *C. albicans* were amplified from the synthesized cDNA in a total reaction volume of 24 μl, using 12 μl of master mix, 1 μl of each of the reverse and forward primers (1 μM), 8 μl of nuclease free water and 2 μl of cDNA not exceeding 100 ng. Relative quantification of the transcripts was performed in a 72-well Rotor-Gene Q series 5 plex (QIAGEN) centrifugal Real-Time Cycler. The PCR parameters were as follows: initial denaturing at 95 °C for 2 min to activate the platinum Taq DNA polymerase, followed by 40 cycles of 5 sec at 95 °C, 30 sec at 60 °C, with fluorescence gain for SYBR green set at 8. A melting curve analysis was generated by the Rotor-Gene software to observe a single melt peak for each sample and to validate that only a single product was present. A PCR run was considered valid when the no template control (NTC) and QuantiTect reverse transcriptase was negative.

**Table 2:** Primer sequences used in real-time PCR transcriptional assay.

Primer Full name	Orientation	Sequence (5′-3′)	Product length (bp)	Region	Reference
*ECE1*	Forward	GTTGCTAATGCCGTCGTC	98	397–494	This study
	Reverse	TCTGGAACGCCATCTCTC			This study
*SIR2*	Forward	TGGACCTGCAACTGGAAC	205	532–537	This study
	Reverse	CCTGCACCAGTAACTACC			This study
*ACT*	Forward	TCCAACTGGGACGATATG	219	183–402	This study
	Reverse	GGTGGTTCTACCAGAAGAG			This study

### Effect of FBS on the ECE1 expression in *C. albicans* biofilm

Biofilm was established and treatment carried out as described above. The tissue culture plate cells were washed in ice-cold sterile PBS and then removed from the plate surface with a sterile scraper. The cells were centrifuged for 20 min at 4 °C and 10000 rpm and the supernatant discarded. The pellets were then lysed with 2 ml of sorbitol lysis buffer and RNA extracted as described above. The test was also performed with 3% FBS in order to measure the effect of FBS on RNA expression. RNA expression was measured as described above.

### Statistical analyses

Two-way ANOVA was carried out for statistical analysis of the inhibitory effects of FGE on biofilm formation in the presence or absence of 3% FBS. The Comparative CT Method (ΔΔCT Method) was used to calculate the fold-differences in *SIR2*, *ECE1* expression. Mean CT values and standard deviations were used in the ΔΔCT calculations. An independent T-test was used to compare *ECE1* expression in two biofilm-associated FBS treated groups. Results were considered significant at *P* ≤ 0.05 and sample CT means, and standard deviations were calculated. The statistical analyses were performed using GraphPad Prism Version 7.04.

### Ethical approval

Ethical approval was not required for this study.
